# Congenital Zika Syndrome: A Nationwide Cohort Study in Brazil, 2015-2018

**DOI:** 10.1056/NEJMoa2101195

**Published:** 2022-02-24

**Authors:** Enny S. Paixao, Luciana L. Cardim, Maria da Conceição N. Costa, Elizabeth B. Brickley, Rita de Cassia Oliveira de Carvalho-Sauer, Eduardo H Carmo, Roberto F. S. Andrade, Moreno S. Rodrigues, Rafael V. Veiga, Larissa C. Costa, Cynthia A. Moore, Giovanny V. A. França, Liam Smeeth, Laura C. Rodrigues, Mauricio L. Barreto, Maria Gloria Teixeira

**Affiliations:** 1 London School of Hygiene and Tropical Medicine, London WC1E 7HT, UK; 2 Center of Data and Knowledge Integration for Health (CIDACS), Gonçalo Moniz Institute, Oswaldo Cruz Foundation, Salvador, Bahia, Brazil; 3 Instituto de Saude Coletiva, Federal University of Bahia, Salvador, Bahia, Brazil; 4 East Regional Health Center of the State Health Secretariat of Bahia, Santo Antonio de Jesus, Bahia, Brazil; 5 Division of Birth Defects and Infant Disorders, National Center on Birth Defects and Developmental Disabilities, Centers for Disease Control and Prevention, Atlanta, GA 30341; 6 Secretariat of Health Surveillance, Ministry of Health, Brasilia, Brazil

## Abstract

**Background:**

Prenatal exposure to ZIKV has potential teratogenic effects with a wide spectrum of clinical presentation called congenital Zika syndrome (CZS). There are limited data on survival of children with CZS, we estimated mortality comparing live births with and without CZS.

**Methods:**

A population-based cohort study using linked routinely collected data in Brazil, from January 2015 to December 2018. Kaplan-Meier and survival analyses were performed adjusted for confounding and stratified by gestational age, birth weight and small for gestational age.

**Results:**

We followed 11,737,554 live births for up to 36 months. The mortality rate among live births with CZS was 52.6 (95% confidence interval [CI] 47.6-58.0) and among those without CZS it was 5.6 (5.6-5.7) per 1000 person-years. The mortality rate ratio was 11.3 (95%CI 10.2-12.4) times higher among live births with CZS than those without CZS up to the age of 36 months. For infants born before 32 weeks' gestation or with birth weight less than 1500g, the risk of death was similar regardless of CZS. Infants with CZS born at term (mortality rate with and without CZS—38.4 vs 2.7) or with birth weight greater than 2499g (mortality rate with and without CZS—32.6 vs 2.5) were 14.3 (95%CI 12.4-16.4) and 12.9 (95%CI 10.9-15.3) times more likely to die than those without CZS. The burden of congenital anomalies, diseases of the nervous system and infectious diseases, as recorded causes of deaths, were higher among the CZS group.

**Conclusion:**

There is a higher mortality risk in live births with CZS than live births without CZS that persists throughout the first three years of life.

Zika virus (ZIKV) infections during pregnancy can be transplacentally transmitted to developing fetuses with potential teratogenic effects, including severe central nervous system anomalies^1^. Although most fetuses with prenatal ZIKV exposure present no detectable clinical anomalies, a subset of congenitally infected offspring can have mild, moderate or severe adverse outcomes^2^ collectively referred to as congenital Zika syndrome (CZS)^3,4^. The CZS phenotype encompasses a wide spectrum of structural anomalies (e.g., cortical atrophy with microcephaly^5^), functional impairments (e.g., dysphagia^6^), and clinical sequelae (e.g., epilepsy^7,8^) that may manifest either at birth or in early life^9^. The prognosis for live births with CZS is not fully described; however, the most severe phenotype seems to be associated with first-trimester exposure^10^ and one important prognostic factor is the severity of microcephaly^11^. Preliminary evidence indicates that affected children may experience a case fatality rate of 10% in the first years of life^12^. Nevertheless, the relative risk of mortality among live-born children with CZS compared to live-born children without CZS and the role of important predictors of child mortality such as gestational age at birth and birth weight remains unknown.

Using national population-based linked data on more than 11.7 million live births born between 2015 and 2018 in Brazil, a country with a significant burden of CZS in the recent ZIKV epidemic, this study aimed to: (i) investigate mortality risk and causes of deaths among live births with CZS compared with those without CZS, (ii) compare mortality rates in live births with CZS with and without microcephaly, and by the timing of maternal rash, and (iii) examine the role of gestational age at birth and birth weight by calculating stratified mortality rate ratios.

## Methods

### Study design

We conducted a retrospective population-based cohort study, including all singletons live births in Brazil from January 1, 2015 to December 31, 2018. These live births were followed-up from birth until December 31, 2018, death, or age of 36 months.

### Data source

We obtained information about CZS from the Public Health Event Record^13^, which registered information from all cases with suspected microcephaly and/or central nervous system (CNS) alterations possibly associated with congenital ZIKV infection since 2015. From the Public Health Event Record, we retained data on final disease classification (confirmed or excluded), maternal rash during pregnancy and live birth head circumference.

The Live Births Information System-SINASC^14^, an information system with 100% coverage of the Brazilian territory^15^, records data from the Declaration of Live Births, a legal document completed by the health worker who assists the delivery. From this system, we obtained information about the mother (maternal age, education, marital status, ethnicity); the pregnancy (antenatal appointments, length of gestation, multiple fetuses); and the newborn (birth weight, sex).

Death-related information was obtained from the Mortality Information System, which records the Death Certificates. The death certificate is a legal document that must be completed by the physician responsible for clinical care, an assistant or another practician from the institution who attests to the cause of death. In cases when the death occurs without medical assistance, the death certificate will be provided by a pathologist. We obtained information on the date and the underlying cause of death by International Classification of Diseases/ICD 10. As of 2011, Mortality Information System was estimated to cover 96.1% of deaths in Brazil^16^.

### Linkage process

Live births records from Live Births Information System were linked with the Public Health Event Record and Mortality Information System. Name, date of birth or age, and maternal residence were used as matching variables. The linkage was performed with the Center of Data and Knowledge Integration for Health -RL-Record Linkage; a novel record-linkage tool developed to link large-scale administrative datasets at Center of Data and Knowledge Integration for Health applying the combination of indexing and searching algorithms approach^17^. All data were extracted in 2020 and made available by the Brazilian Ministry of Health. Linkage procedures were conducted at Center of Data and Knowledge Integration for Health in a strict data protection environment and according to ethical and legal rules^18^.

### Procedures

We included all singleton live births who contributed records during the study period. We excluded records registered in the Public Health Event Record as suspected of CZS but ruled out after epidemiological investigation and those classified as cases under investigation or inconclusive.

In Brazil, live births who meet one or more of the following criteria should be reported and investigated as suspected cases of CZS: 1) microcephaly defined as an head circumference more than 2 standard deviations below the mean for age and sex (according to INTERGROWTH 21^st^ standards ^19^ for those born with less than 37 gestation weeks, or World Health Organization (WHO) standards for those born with 37 gestation weeks or more); 2) craniofacial disproportion (microcrania in relation to the face); CNS changes suggestive of congenital infection detected from neuro-imaging tests (accepted imaging were cranial computed tomography, brain magnetic resonance, or transfontanellar ultrasound); two or more neurological, visual or auditory manifestations; 3) newborns or fetuses from mothers who reported a fever and/or skin rash during pregnancy, likely or confirmed for ZIKV infection, regardless of the identification of congenital malformations at birth^20,21^. After notification, all suspected cases were investigated by the epidemiological surveillance teams and classified as confirmed, probable, inconclusive or excluded cases (Figure S2).

Suspected cases were considered confirmed when they had signs and symptoms consistent with CZS (Table S2), and laboratory evidence of ZIKV infection (from molecular or serological testing) or their mother reported fever and/or rash during pregnancy. Probable cases showed clinical changes compatible with CZS, and tested negative for other congenital infections, but the specific laboratory diagnosis for ZIKV infection was not available, and the mother was asymptomatic during pregnancy. Suspected cases were considered excluded if they had compatible clinical symptoms that, after clinical and epidemiological investigation, were attributed to having another cause, for example, microcephaly related to restricted intrauterine growth or genetic diseases. Other cases were inconclusive due to insufficient information for proper classification or remained under investigation^20^.

We defined CZS cases as all live births classified in the Public Health Event Record as confirmed or probable cases and had a register linked with a Live Births Information System record. We then classified live births with CZS into two categories: with microcephaly (head circumference more than 2 standard deviations below the mean for age and sex) or without microcephaly, according to INTERGROWTH 21^st^ standards. We also classified CZS cases according to the time of maternal rash during pregnancy (first trimester, second trimester, third trimester, or no report of rash during pregnancy). Live births that died during the study period were identified by linking the Live Births Information System with Mortality Information System.

### Statistical analyses

Descriptive statistics are presented for maternal sociodemographic data and newborn characteristics. Mortality rates (deaths/1,000-person-year (PY) and crude hazard ratios (HRs) with 95% CIs comparing live births with CZS to live births without CZS were estimated using Cox proportional hazards models. We also conducted a sensitivity analysis with confirmed cases only. Kaplan-Meier curves were plotted, and we compared live births with and without CZS in total, CZS according to head circumference (classified as with microcephaly and without microcephalic) and CZS according to maternal rash during pregnancy compared with live births without CZS. Finally, we fitted penalized Cox proportional hazards regression models using restricted maximum likelihood with frailty terms corresponding to random effects from a Gaussian distribution to account for within-cluster (maternal residency region) homogeneity in outcomes^22^. We used these analyses to obtain the adjusted HR. The adjusted models were controlled for maternal age, education, marital status, ethnicity, number of prenatal appointments, newborn sex and year of birth and stratified by gestational age (< 32, 32-36, ≥37 weeks), birth weight (<1500, 1500-2499, ≥2500 grams) and small for gestational age (smaller than the 10th centile according to the INTERGROWTH 21^st^ standards). For the time scale in our survival analyses, we used age attained within the study, neonatal mortality (up to 27 days), infant mortality (up to 364 days) and mortality up to 36 months. Data analyses were performed in Stata version 15.0.

This study analyzed de-identified data and was approved by the Institute of Collective Health, Federal University of Bahia Research Ethics Committee (CAAE registration no. 70745617 2 0000 5030).

## Results

We followed 11,737,554 live births from birth up to 36 months (mean 23 [range 0–36]) (Figure S1). The characteristics of our study population are reported in Table 1. In general, live births with CZS (n=3,308) had mothers who were younger and less educated. Nearly 20% of the live births with CZS were born preterm, 36% were low birth weight (LBW), and 37% were small for gestational age (SGA) compared with 10.2% preterm births, 7.3% LBW and 6.9% SGA among live births without CZS (n=11,477,907).

By the end of the study period, 398 live births who met the study case definition criteria of CZS and 120,629 live births without CZS had died. The overall mortality rate up to 36 months in live births with CZS was 11.3 times higher (95% CI, 10.2-12.4) than in live births without CZS. The increased risk of death persisted throughout the observation period with no suggestion of attenuation by the age of three years in children with CZS. The highest mortality rate ratio, 21.9 (95% CI, 17.3-27.6), was found after the first year of life when the mortality in children without CZS was 0.7 deaths/1000 PY (Table 2). The analysis restricted to confirmed CZS cases showed similar results (Table S1).

**Figure 1:**
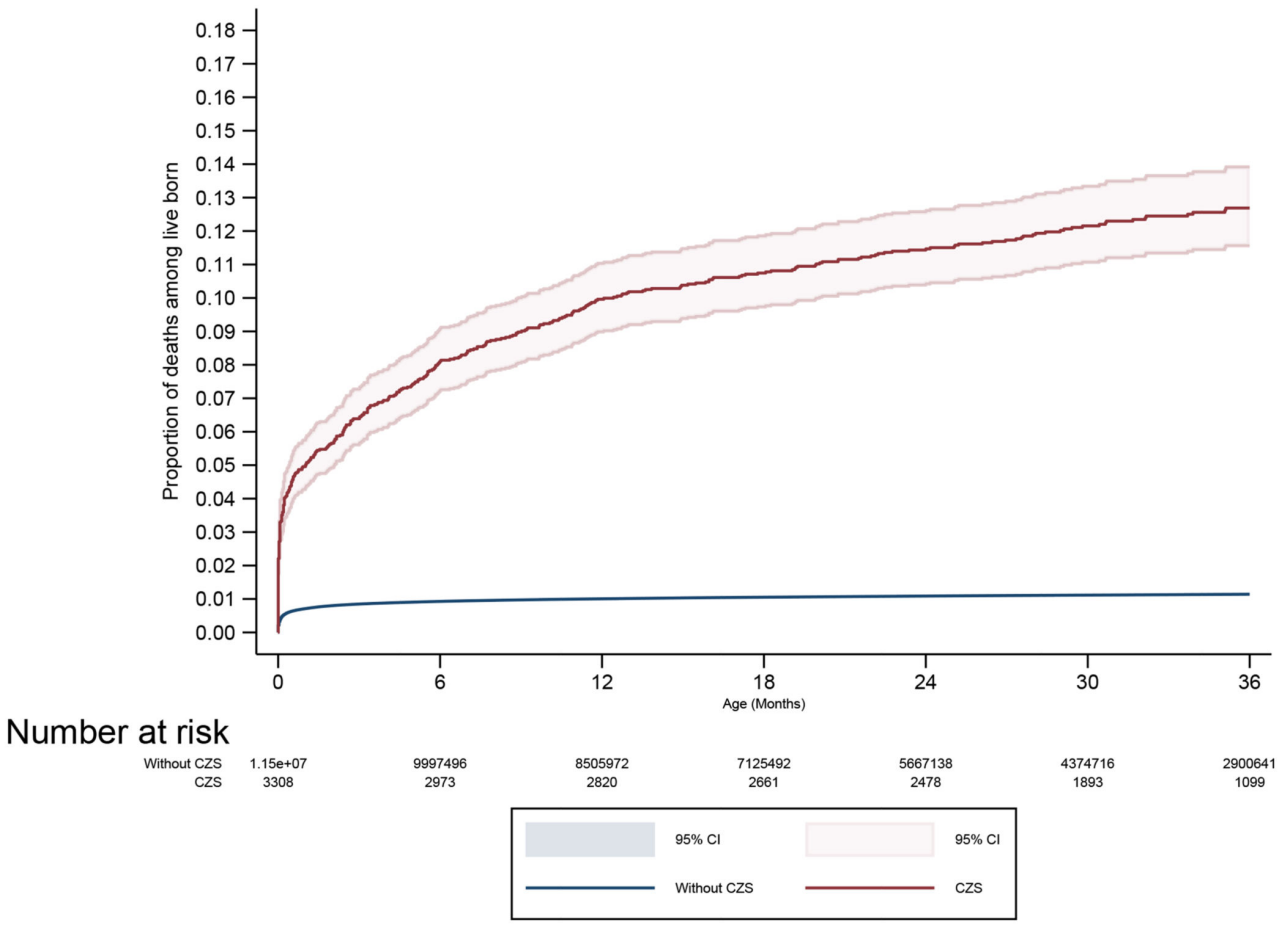

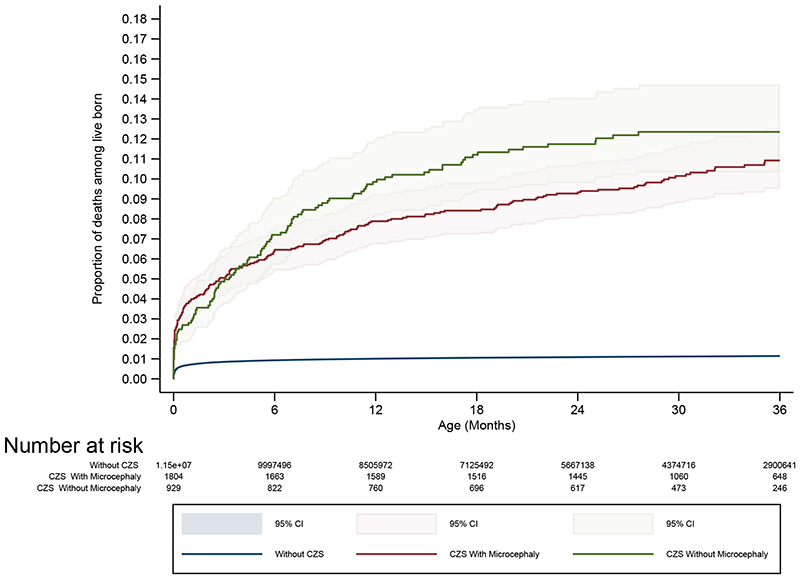

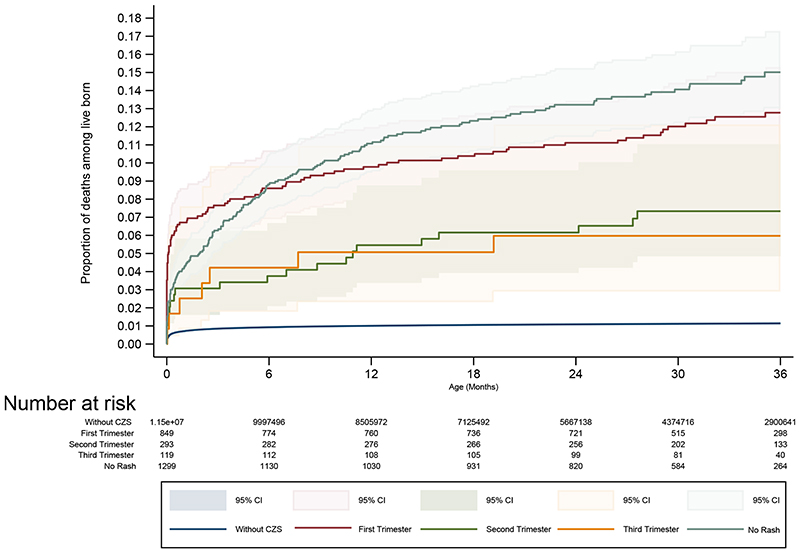
Kaplan-Meier curves showing survival comparing CZS births with non-CZS births up to 36 months of age (A), stratified by head circumference (B) and maternal rash (C).

The likelihood of death in the study population from birth up to 36 months is seen in Figure 1. Among live births with CZS the probability of death was greater than in live births without CZS, and the difference continued to increase during the study period. Information on head circumference was available for 82.6% (2,733/3,308) of live births with CZS: 66.0% had microcephaly at birth. Among live births with CZS and complete information on maternal rash, 51.2% (1,364/2,663) reported this symptom during pregnancy, with 62.2% of exanthems reported in the first trimester, 21.5% the second, 8.7% the third and 7.6% with unspecified trimester. The association of CZS on mortality up to 36 months did not materially differ for live births with and without microcephaly or for whether mothers reported a rash during pregnancy and the timing of any rash. However, numbers were relatively small, and we may have lacked the power to identify sub-group differences (Figure 1).

**Figure 2:**
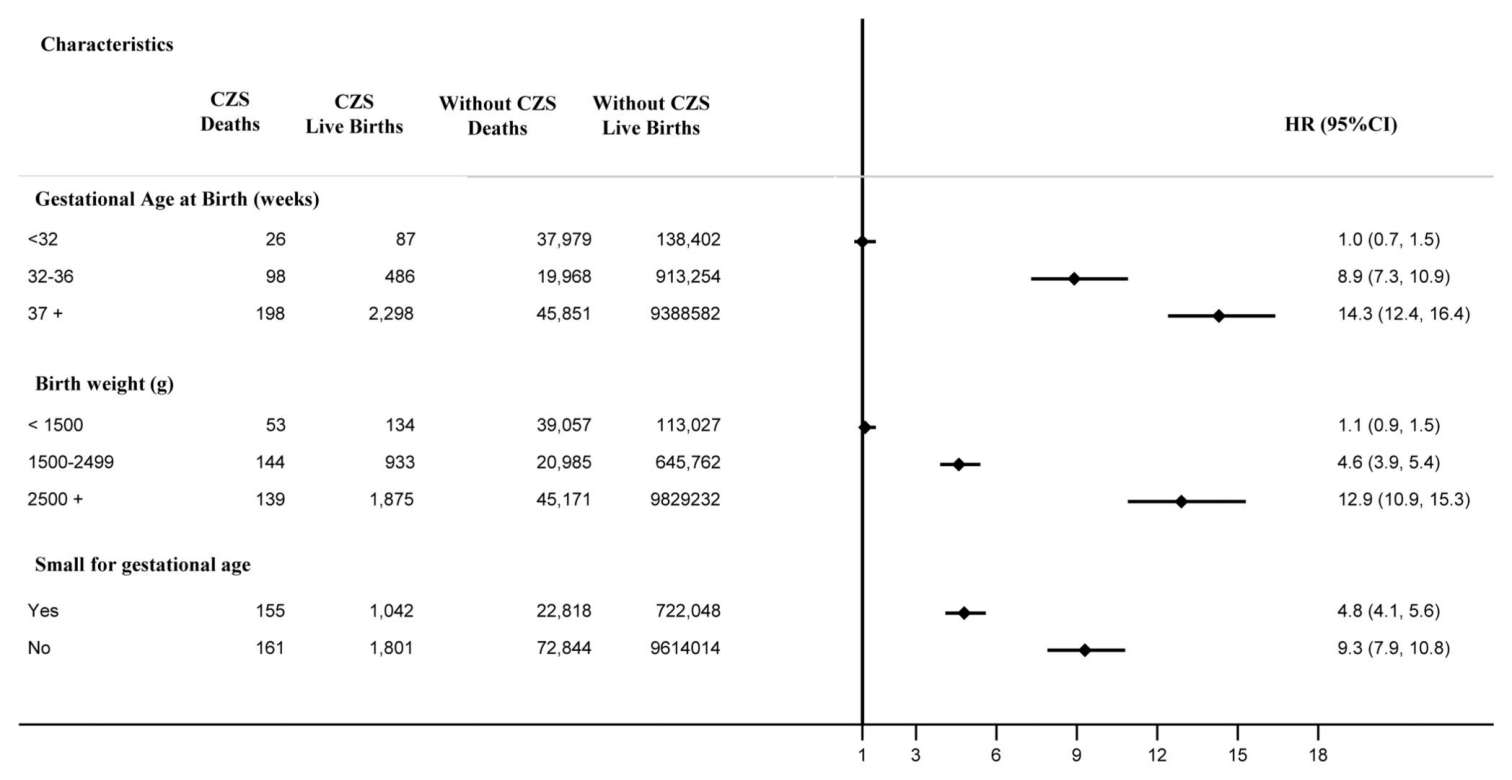
Cox proportional hazards for mortality risk comparing CZS births with non-CZS births up to 36 months of age stratified by gestational age at birth, birth weight and small for gestational age *CZS: Congenital Zika Syndrome; HR: Hazard Ratio; CI: Confidence Interval * adjusted by maternal age, education, and marital status, ethnicity, number of prenatal appointments, new born sex and year of birth

Mortality rates differ across gestational age, birth weight and small for gestational age categories. Live births with and without CZS had similar absolute mortality rates if they were born before 32 weeks or weighed less than 1500g. If they were born after 32 weeks, live births with CZS were more likely to die than live births without CZS, and the highest ratios were observed among children born term and with normal birth weight; in these groups, live births with CZS were 14.3 (95% CI, 12.4-16.4) and 12.9 (95% CI, 10.9-15.3) times more likely to die when compared with their counterpart live births without CZS, respectively. Small for gestational age live births with CZS were almost five times more likely to die, while appropriate for gestational age children were nine times more likely to die than their counterpart live births without CZS (Figure 2).

**Figure 3:**
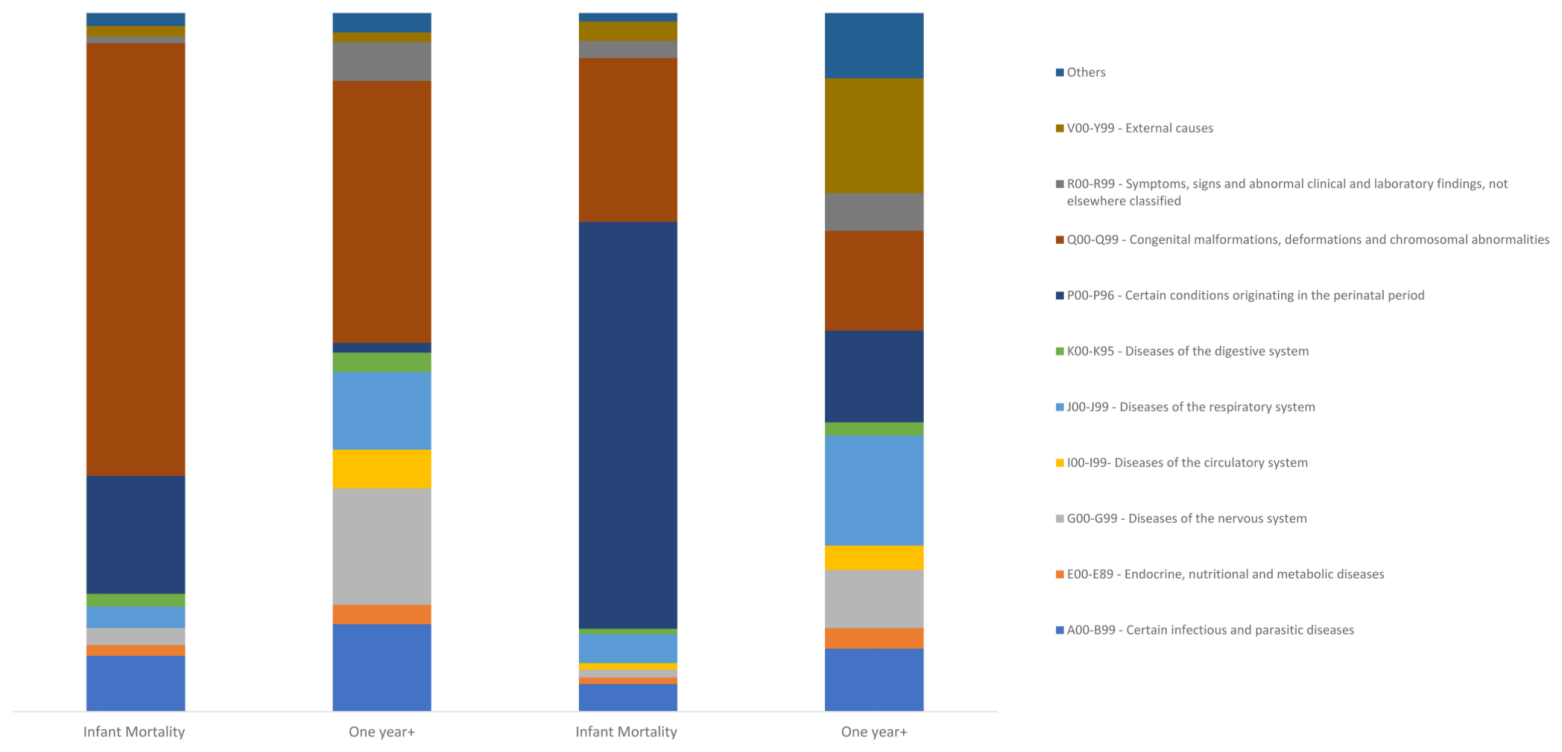
Causes of death using ICD-10 chapter according to age group by CZS status Brazil, 2015-2018. CZS: Congenital Zika Syndrome

Among infants with CZS, the causes of deaths coded under ICD-10 certain infectious and parasitic diseases (A00-B99), diseases of the nervous system (G00-G99), and congenital malformations (Q00-Q99) were two times more common than among infant without CZS. The leading causes were sepsis, unspecified organism; hydrocephalus unspecified; and microcephaly, respectively. After the first year of life, the nervous system diseases and congenital anomalies continue to be leading chapters. However, for the G00-G99 chapter, the most common cause of death in this age group was cerebral palsy. The circulatory system diseases (I00-I99) were responsible for 58% more deaths in the CZS group than live birth without CZS (Figure 3). The causes of deaths were cardiomyopathy, other cardiac arrhythmias, and heart failure.

## Discussion

Analyses of Brazilian national, registry-based data showed that the mortality rates up to 3 years old were more than eleven times greater in live births with CZS than live births without CZS overall. Among live births with CZS, mortality risk by microcephaly status and maternal reported rash did not materially differ. The risk of death among the smallest infants was similar regardless of CZS status; however, term and normal birth weight live births with CZS were over 12 times more likely to die than their counterparts without CZS. Finally, we observed that conditions classified as congenital malformations, diseases of the nervous system and certain infectious diseases were more common causes of deaths among live births with CZS than those without CZS.

Data on mortality associated with CZS are scant. In a series of confirmed CZS cases in Brazil, mortality risk up to 8 days was estimated at 41.1 per 1000 live births^23^. In a population-based surveillance study conducted among all infants and fetuses with congenital abnormalities potentially related to Zika virus infection in the USA, the neonatal mortality risk was 45.9 per 1000 live births^24^. A similar risk was observed in our study. However, only a longer follow-up could have revealed that after the neonatal period the absolute mortality rates in the live births with CZS did not decrease as dramatically over time as they did among live births without CZS.

Our understanding of the impact of CZS on the brain is still emerging; the effects of CNS dysfunction with or without microcephaly are expected to result in a wide variety of outcomes^4,25^. Although some studies have shown that the most severe phenotype appears to be associated with exposure during the first trimester^25,26^, neither early exposure (using rash as a proxy) nor microcephaly status showed difference in the mortality risk, potentially due to the small number of events. Therefore, the role of head circumference and timing of maternal symptoms of ZIKV infection as risk factors for death among children with CZS cannot be fulled assessed.

Previous studies have shown that live births with CZS had greater frequencies of LBW^27^ and SGA^28,29^, features compatible with higher child mortality. However, the absolute mortality risks in the smallest infants did not differ according to CZS status. Moreover, in the CZS group, term normal-weight infants, who would have had a high chance of thriving without the impairments resulting from CZS, are at strikingly elevated mortality risk. Children with CZS have multiple neurological complications and long-term sequelae, such as cerebral palsy that was one of the main causes of deaths identified in this study and epilepsy, estimated in 67% in this group^30^, that confer an increased risk of death. However, a better understanding of the causal mortality chain is needed.

A strength of our study was the large sample size, including all confirmed and probable CZS cases notified in the country. We also included a population-representative comparison group and were able to control for confounding. The sensitivity analyses (including only confirmed cases) showed consistency of our findings. There are, however, limitations. First, the present study was based on registry data, and relevant clinical data were not available. Second, at the beginning of the epidemic, the health services network did not have specific diagnostic tests for ZIKV infections. Therefore, there may have been underreporting in the Public Health Event Record, mainly among those fetuses prenatally exposed to ZIKV, but without detectable malformations at birth. Third, the linkage process could have introduced classification bias due to a linkage error. However, if an error occurred in the linkage that enabled the exposure assessment (Public Health Event Record) it would have likely underestimated the measure of association. If the error occurred in the linkage that enabled the outcome assessment (Live Births Information System - Mortality Information System) it is probably non-differential and unlikely to introduced bias in the measure of association, although the absolute measures of risk may be underestimated. There was a slight variation in data completeness by CZS status. However, data on all variables were more than 80% complete in both groups.

Our findings draw attention to the importance of primary prevention of infection in women (of childbearing age and pregnant women) against *Aedes aegypti* bites.

Disclosure forms provided by the authors are available with the full text of this article at NEJM.org.

## Supplementary Material

Supplement

## Figures and Tables

**Table 1:** Baseline characteristics (n and %) of singleton live births in the cohort-linked data by congenital Zika syndrome status, Brazil 2015-2018.

Characteristics	With CZS		Without CZS	
	n	%	N	%
**Maternal age (years)**				
< 20	769	23.2	1,958,784	17.1
20-34	2,179	65.9	7,922,774	69.0
35+	360	11.9	1,596,199	13.9
**Maternal marital status**				
Single/Widow/Divorced	1,736	53.1	5,046,501	44.0
Married/union	1,532	46.9	6,303,447	54.9
**Maternal education (Years)**				
none	25	0.8	54,922	0.5
1 - 3	106	3.3	264,514	2.3
4 – 7	749	23.0	1,932,786	16.8
8 – 12	2,011	61.8	6,834,566	59.5
12 +	365	11.2	2,225,266	19.4
**Maternal race/ethnicity**				
White	584	18.8	3,987,857	36.4
Black, Mixed and others	2,517	81.2	6,973,896	63.6
**Number of prenatal appointments**				
None	51	1.6	64,992	0.6
0-3	307	9.7	683,661	6.0
4 – 6	923	29.1	2,609,078	22.7
7+	1,895	59.7	7,824,331	68.2
**Year of birth**				
2015	1.215	36.7	2,945,913	25.7
2016	1538	46.5	2,794,266	24.3
2017	364	11.0	2,857,930	24.9
2018	191	5.8	2,879,798	25.1
**Sex of the newborn**				
Female	1,757	53.2	5,591,949	48.7
Male	1,543	46.8	5,883,957	51.3
**Birth region**				
North	181	5.5	1,237,863	10.8
Northeast	1,986	60.0	3,224,654	28.1
Southeast	801	24.2	4,508,232	39.3
South	73	2.2	1,553,930	13.5
Midwest	265	8.0	949,767	8.3
**Gestational age at birth (weeks)**				
<32	110	3.4	155,151	1.4
32-36	535	16.7	988,036	8.8
37 +	2,552	79.8	10,085,776	89.8
**Birth weight (g)**				
< 1500	155	4.7	127,967	1.1
1500-2499	1,048	31.7	706,939	6.2
2500+	2,103	63.6	10,632,209	92.7
**Small for gestational age**				
No	2,000	63.4	10,317,503	89.9
Yes	1,156	36.6	794,313	6.9

* 896,987 missing data

**Table 2:** Mortality risk by age group among singleton live births in the cohort-linked data, Brazil, 2015-2018.

Live births	Live births with CZS	Live births without CZS	HR for mortality (95% CI)
**Neonatal mortality (up to 27 days)**			
Neonatal deaths	163	80,006	7.2 (6.2-8.4)
Deaths per 1,000 PY	696.5 (597.3 -812.0)	95.7 (95.0 - 96.3)	
**Post-neonatal mortality (28-364 days)**			
Post-neonatal deaths	163	32,175	17.4 (14.9-20.3)
Deaths per 1,000 PY	59.4 (51.0 - 69.3)	3.5 (3.5 - 3.6)	
**Infant mortality (up to 364 days)**			
Infant deaths	326	112,181	10.2 (9.1-11.3)
Deaths per 1,000 PY	109.2 (98.0 - 121.8)	11.3 (11.2 - 11.3)	
**Mortality after one year (12-36 months)**			
Deaths after 1 year	72	8,448	21.9 (17.3-27.6)
Deaths per 1,000 PY	15.7(12.4-19.7)	0.7 (0.7-0.8)	
**Total mortality (up to 36 months)**			
Total Deaths	398	120,629	11.3 (10.2-12.4)
Deaths per 1,000 PY	52.6 (47.6-58.0)	5.6 (5.6-5.7)	

PY= person years CZS = congenital Zika syndrome
