# Supplementary material for: Congenital Zika Syndrome: A Nationwide Cohort Study in Brazil, 2015-2018
Source: N Engl J Med. Author manuscript; Available in PMC 2022 Feb 28. (PMC7612437; doi:10.1056/NEJMoa2101195)
Supplement: Supplement [file EMS140779-supplement-Supplement.pdf]

# Appendix

## Congenital Zika Syndrome: A Nationwide Cohort Study in Brazil, 2015-2018

Enny S. Paixao, Ph.D[1,2*], Luciana L. Cardim, Ph.D[2*], Maria da Conceição N. Costa, MD, Ph.D[2,3], Elizabeth B. Brickley, Ph.D[1], Rita de Cassia Oliveira de Carvalho-Sauer, MSc[4], Eduardo H Carmo, MD, Ph.D[2], Roberto F. S. Andrade, Ph.D[2], Moreno S. Rodrigues, Ph.D[2], Rafael V. Veiga, Ph.D[2] , Larissa C. Costa, Ph.D[2] Cynthia A. Moore, MD, Ph.D[5], Giovanny V. A. França, Ph.D[6], Liam Smeeth, MD, Ph.D[1] Laura C. Rodrigues MD, Ph.D[1,2], Mauricio L. Barreto, MD, Ph.D[2,3#], Maria Gloria Teixeira MD, Ph.D[2,3#]

**Table of contents**

**Figure S1: Flowchart study population Brazil, 2015-2018 ---------------------------------------------2**

**Figure S2 Flowchart for classification criteria for congenital Zika syndrome ---------------------3**

**Table S1: Mortality risk by age group among singleton live births in the cohort-linked data (CZS confirmed cases only), Brazil, 2015-2018. ----------------------------------------------------------------------------------------------4**

**Table S2: Main findings in infants with congenital Zika syndrome --------------------------------5**

**Figure S1. Flowchart study population Brazil, 2015-2018.**

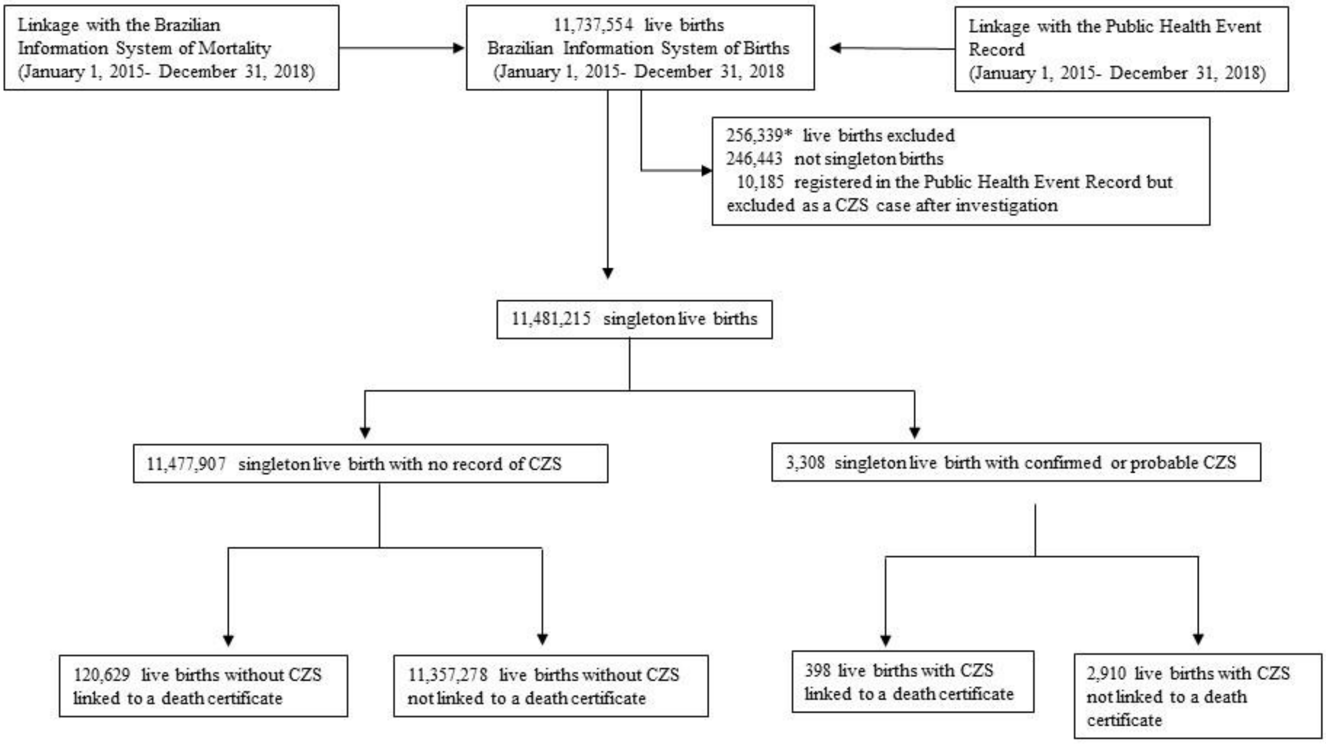


* The exclusions do not sum to 256,339 because a record may be multiples and excluded as a CZS case after investigation

• Congenital Zika Syndrome

**Figure S2. Flowchart for classification criteria for congenital Zika syndrome**

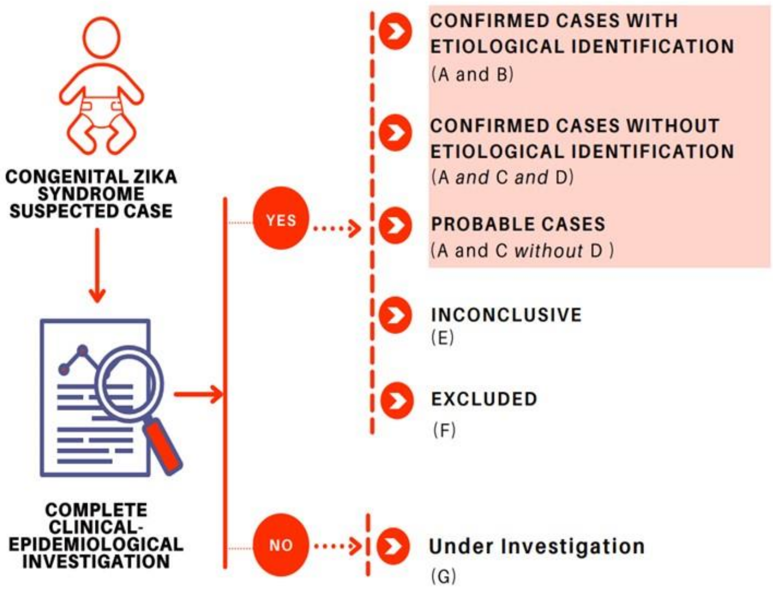


* Main findings related to congenital Zika syndrome are described in supplementary table S2

**A** – two or more signs and symptoms of the main findings related to congenital Zika syndrome in an image or clinical examination, as described in supplementary table S2;

**B** – positive result for ZIKV infection in a molecular (RT-PCR) or serological test of a sample of the mother, fetus, or newborn (in the first 48 hours of life);

**C** – negative or inconclusive result for ZIKV infection in a molecular (RT-PCR) or serological test of a sample of the mother, fetus, or newborn (in the first 48 hours of life), or test not performed;

**D** – maternal report of rash or fever during pregnancy;

**E** – clinical-epidemiological investigation not completed due to refusal or because it is impossible to find the children after three or more attempts and whose results and available information do not allow it to be classified. ;

**F** – clinical-epidemiological investigation led to the diagnosis of another etiology or was not compatible with the classification criteria for confirmed, probable or inconclusive cases of congenital zika syndrome;

**G** – clinical-epidemiological investigation so far has not yet reached enough information to enable diagnostic classification.

***Based on the Brazilian Ministry of Health Guideline**

**Table S1: Mortality risk by age group among singleton live births in the cohort-linked data (CZS confirmed cases only), Brazil, 2015-2018.**

| Live births | Live births with CZS | Live births without CZS | HR for mortality (95% CI) |
|---|---|---|---|
| **Neonatal mortality (up to 27 days)** | | | |
| Neonatal deaths | 123 | 80,006 | |
| Deaths per 1,000 PY | 639.01 (535.50-762.54) | 95.7 (95.0 - 96.3) | 6.6 (5.5-7.9) |
| **Post-neonatal mortality (28-364 days)** | | | |
| Post-neonatal deaths | 119 | 32,175 | |
| Deaths per 1,000 PY | 51.98 (43.43-62.21) | 3.5 (3.5 - 3.6) | 15.3 (12.8-18.3) |
| **Infant mortality (up to 364 days)** | | | |
| Infant deaths | 242 | 112,181 | |
| Deaths per 1,000 PY | 97.3 (85.7-110.3) | 11.3 (11.2 - 11.3) | 9.2 (8.1-10.4) |
| **Mortality after one year (12-36 months)** | | | |
| Deaths after 1 year | 57 | 8,448 | |
| Deaths per 1,000 PY | 14.01 (10.9-18.3) | 0.7 (0.7-0.8) | 19.8 (15.2-25.7) |
| **Total mortality (up to 36 months)** | | | |
| Total Deaths | 299 | 120,629 | 10.2 (9.1-11.5) |
| Deaths per 1,000 PY | 45.8 (40.9-51.3) | 5.6 (5.6-5.7) | |

PY= person years

*****Confirmed cases only

**Table S2: Main findings in infants with congenital Zika syndrome (translation from material published by the Brazilian Ministry of Health)[1]**

| **Most common findings identified prenatally** | | | |
|---|---|---|---|
| ***Structural CNS/cranial*** | ***Facial dysmorphia*** | ***Neurologic sequelae*** | ***Other*** |
| Microcephaly/microencephaly | Flat face | Static position of hands and feet (indicative of arthrogryposis) | Polyhydramnios |
| Craniofacial disproportion | Retrognathia | | |
| Prominent occipital bone | Hypotelorism | | |
| Misshapen cerebellar vermis | Redundant scalp | | |
| Ventriculomegaly (mild, moderate, severe), ex vacuo/hydrocephalus | | | |
| Brain calcifications, diffuse | | | |
| Ventricular synechiae | | | |
| Agenesis of the corpus callosum | | | |
| Schizencephaly/porencephaly | | | |
| Cerebral cortex thinning | | | |
| Microphthalmia | | | |
| **Most common findings identified at birth or within the first month of life** | | | |
| ***Structural CNS/cranial*** | ***Neurosensory sequelae*** | ***Neurologic sequelae*** | ***Other*** |
| Microcephaly | Retinal mapping changes | Changes in muscle tone | |
| Craniofacial disproportion | Lesions of retinal epithelium/ unusual pigmentation | Changes in posture | |
| Brain calcifications | Retinal atrophic circular lesions | Hyperexcitability/hyperirritability | |
| Abnormal cortical development/ polymicrogyria/simplified gyri | Optic nerve changes (hypoplasia, partial/complete atrophy, increased papillary excavation) | Epileptic seizures | |
| Cortical thickening, frontoparietal | Alteration of visual function | Difficulties with suck and swallow | |
| Ventriculomegaly | Alteration of auditory function | Joint contractures | |
| Hypoplastic brain stem | Abnormal eye movements | | |
| Hypoplastic cerebrum/alterations posterior fossa | | | |
| Hypoplastic corpus callosum | | | |
| Retinal and optic nerve anomalies | | | |
| **Findings identified after the first month of life** | | | |
| ***Structural CNS/cranial*** | ***Neurosensory sequelae*** | ***Neurologic sequelae*** | ***Other*** |
| **Most frequent** | | | |
| Craniofacial disproportion | Visual changes (inattention) | Dysphagia | Congenital hip dislocation |
| Changes in HC due to hydrocephalus | Strabismus, nystagmus | Epilepsy/spasms | |

| | | | |
|---|---|---|---|
| Brain calcifications | Changes in retinal mapping | Irritability | |
| Dysmorphic corpus callosum | Alteration of auditory function | Hypertonia | |
| Ventriculomegaly | | Persistent primitive reflexes | |
| **Frequent** | | | |
| | Sensorineural hearing loss | | |
| **Rare** | | | |
| Microphthalmia | | | Cryptorchidism |
| Coloboma | | | Hypospadias |
| | | | Cataract |
| | | | Glaucoma |

CNS – central nervous system
HC – head circumference

## Reference


1. Ministério da Saúde (BR). *Orientações integradas de vigilância e atenção à saúde no âmbito da Emergência de Saúde Pública de Importância Nacional*. *Ministério da Saúde.* (2017).
